# Bridging Classrooms and Communities: The Transformative Impact of Community‐Based Dental Education on the Learning Experiences of Undergraduate Students

**DOI:** 10.1111/eje.13076

**Published:** 2025-01-27

**Authors:** Kamran Ali, Sruthi Sunil, Nidhi Gupta, Rebecca Glanville, T. Vanishree, Sadeq Ali Al‐Maweri, Asmaa Al Khtib

**Affiliations:** ^1^ QU Health College of Dental Medicine Qatar University Doha Qatar; ^2^ Peninsula Medical School, Faculty of Health University of Plymouth Plymouth UK; ^3^ Century International Institute of Dental Sciences and Research Center Poinachi Kerala India; ^4^ Qatar University College of Dental Medicine Doha Qatar

**Keywords:** community‐based dental education, dental, students, undergraduate

## Abstract

**Aims:**

This study aimed to evaluate the impact of community‐based dental education (CBDE) on the learning experiences of undergraduate dental students and recent dental graduates from two diverse geographical regions.

**Methods:**

The study followed a cross‐sectional design, conducted online using Google Forms, with ethical approval from Qatar University. A non‐probability purposive sampling method was used to recruit dental students and recent graduates from three institutions in India and one in Qatar. A questionnaire based on 20 closed‐ended items and two open‐ended questions, developed by a team of dental academics, was used for data collection. Data analysis involved descriptive statistics, analysis of variance (ANOVA) and thematic analysis for open‐ended responses.

**Results:**

The study included 148 participants, with 116 female (78.37%) and 32 male (21.62%) students, mostly from India (75%) and the rest from Qatar (25%). ANOVA revealed significant differences based on age, country and stage of education (*p* < 0.001). Dental interns had the highest mean score (1.12 ± 0.8), while Year 2 students had the lowest (0.75 ± 1.08). Female participants had a slightly higher mean score (1.05 ± 0.77) than male participants (0.92 ± 0.98), although this difference was not statistically significant (*p* = 0.127). Key challenges included inadequate support and cultural barriers in India, and conflicts with religious obligations and didactic workload in Qatar. Recommendations focused on enhancing field activities, logistical support and mentorship programmes.

**Conclusions:**

The study shows that dental students value CBDE for developing essential skills for community engagement. However, challenges differ across socio‐cultural contexts, highlighting the need for more localised and supportive frameworks to improve CBDE experiences. The variation in student perceptions by age, education stage and country suggests that CBDE strategies should be flexible and adaptable to address the diverse learning needs of students.

## 1 Introduction

Community‐based dental education (CBDE) is a well‐established approach in dental training, integrating academic goals with community service to foster a more holistic learning experience [1, 2, 3]. Its importance is underscored by the fact that traditional dental education models differ significantly from their medical counterparts; dental schools typically own and operate their patient care clinics, primarily emphasising student education [2]. CBDE enriches students' learning experiences and provides essential dental care to underserved populations [3]. By moving part of the education from conventional academic settings into real‐world community environments, CBDE enables students to enhance their clinical skills, cultural awareness and understanding of public health issues [2]. Students tackle diverse oral health challenges through hands‐on experience in underserved areas and apply theoretical knowledge in practical, community‐based settings [2, 4].

The impact of CBDE extends far beyond improving clinical competency. It fosters a sense of social responsibility and civic engagement, as students are exposed to the complexities of providing care to populations with limited access to dental services [5]. This exposure broadens their perspective on healthcare disparities and instils a commitment to serving vulnerable communities [6]. In addition to these social benefits, community‐based experiences prepare students to work in interdisciplinary teams, collaborate with public health initiatives and adapt to varied healthcare environments [6]. CBDE aligns with the Commission on Dental Accreditation (CODA) standards, which require dental education programmes to provide opportunities for community‐based learning [7, 8]. Research indicates that students trained in these environments exhibit higher clinical productivity, improved confidence and greater competence than those trained exclusively in traditional settings and tend to experience a smoother transition into professional dental practice [7, 8, 9, 10]. Evaluations of these programmes show that students not only improve their clinical skills but also integrate theoretical knowledge with practical application, gaining a deeper understanding of the social, ethical and cultural aspects of oral health within the community [5].

Challenges such as language barriers, anxious patients and building rapport in community settings are commonly faced by students, but these difficulties also highlight the importance of communication skills in fostering effective provider–patient relationships [5]. Dental students who recognised the importance of communication skills in practice reported gaining more from their learning experiences [11]. CBDE encourages students to refine these skills and develop cultural awareness, which is essential for improving patient cooperation and ensuring ethical, patient‐centred care [5, 12]. Enhancing students' cultural awareness early in their education reduces barriers to dental services and enhances their ability to serve diverse populations [13].

One of the key components of CBDE is guided reflection, which helps students explore their assumptions, values and experiences while applying their knowledge in real‐world contexts [14, 15]. This reflective practice strengthens problem‐solving abilities and fosters greater empathy, communication skills and self‐confidence, prompting students to reconsider preconceived notions about oral healthcare. Community‐based experiences have been shown to boost students' confidence in providing clinical care and managing patients, enhance their technical skills and improve their efficiency. Many dental schools around the world currently offer training programmes in community‐based settings outside the dental school environment [3, 16].

This study aimed to evaluate the impact of CBDE on the learning experiences of undergraduate students and recent graduates from two diverse geographical regions. By comparing the learning experiences of participants from different universities, the objective of this study was to provide insights into how CBDE shapes the professional development of dental students and graduates and prepares them for their future roles in the healthcare sector.

## 2 Methods

### 2.1 Ethical Considerations

Ethical approval was obtained from the Institutional Review Board of Qatar University (reference number: QU‐IRB 1877‐EA/23, dated 25 May 2023). Participation in the study was voluntary, and all participants were required to provide informed consent prior to responding to the study questionnaire.

### 2.2 Study Design and Study Setting

This study was based on a cross‐sectional analytical design. The study was conducted online using Google Forms.

### 2.3 Sampling Technique and Participants

A non‐probability purposive sampling technique was used to recruit dental students and recent graduates at three institutions in India and one institution in Qatar.

The Bachelor of Dental Surgery (BDS) programme at the three dental colleges in India extends over 5 years. CBDE activities commence in the third year of the dental programme, and the students receive didactic teaching on epidemiology, community oral health promotion and disease prevention. Outreach community activities commence in the fourth year and continue until the fifth year. During these outreach placements, students engage with local communities, focusing on disadvantaged and underserved population groups. The students gain experience in providing oral health education, conduct oral examinations and provide preventive treatments such as application of fluorides, fissure sealants and basic emergency dental care in community health centres using mobile dental units.

In contrast, the Doctor of Dental Medicine (DDM) programme in Qatar spans over 6 years. CBDE is introduced in Year 3, and students receive didactic teaching in community and public health dentistry and provide oral health education in community settings. These CBDE activities continue in Year 4 and Year 5. In Year 6, students gain clinical experience in community settings during their primary care placements. The students provide comprehensive clinical dental services encompassing the core dental disciplines, including operative dentistry, periodontology, endodontics and oral surgery.

Invites to participate in the research were sent by email to potential participants using professional channels in the participating institutions. The eligibility criteria for participation included full‐time undergraduate students and recent dental graduates who have experienced CBDE in their undergraduate dental programme, along with a participant information sheet explaining the purpose and scope of the study.

### 2.4 Research Instrument

A questionnaire consisting of 20 closed‐ended questions and two open‐ended items was developed by the research team comprising five experienced dental academics. The questionnaire was piloted with 15 undergraduate dental students to determine the relevance, language and clarity of the questionnaire. Pearson correlation (*r* = 0.76) showed satisfactory correlations between continuous variables, while Kendall's tau showed satisfactory correlations between ranked variables (τ = 0.79). Following the pilot, minor amendments were made to improve the language and clarity of four items, and the survey questionnaire was finalised with consensus amongst the research team.

The quantitative questionnaire comprised 20 items based on four key domains. Items 2d and 2e were negatively phrased and so negatively scored with ratings from 2 (for strongly disagree) to −2 (for strongly agree). All other items were positively phrased and so positively scored with ratings from −2 (for strongly disagree) to 2 (for strongly agree) with 0 as the neutral score. The open‐ended section included two items. The first asked the participants to share any barriers or challenges experienced by the participants in CBDE activities at their institution, and the second question asked the participants to provide their recommendations to further enhance the learning experiences of students in CBDE.

### 2.5 Data Collection

The participants were invited to complete an online questionnaire using Google Forms. Prior to accessing the questionnaire, each participant was required to sign a mandatory electronic consent form to confirm they understood the purpose of the study; their participation was voluntary; and that the data were processed anonymously. The participants were also asked to confirm that this was the first time they were providing their responses to prevent multiple responses. Data were collected from 1 August to 30 October 2023. Reminders were sent 2 weeks after the initial invites.

### 2.6 Data Analysis

Descriptive statistics including confidence intervals were calculated for each closed‐ended item and the combined dataset. Analysis of variance (ANOVA) was used to determine any significant variation between the results by gender, stage of study and university. Estimated marginal means were calculated from the ANOVA outcomes. All data were analysed and visualised using RStudio (version 2023.06.2) incorporating R version 4.0.5 [17].

A thematic analysis was undertaken to analyse the responses to the two open‐ended questions. First, the responses were gathered and thoroughly reviewed multiple times to develop a deep understanding of the content. Then, concise labels were assigned to code segments of text that captured particular viewpoints, experiences, or suggestions. Afterwards, these codes were grouped into potential themes by clustering related codes that illustrated broader patterns. Through repeated refinement, the themes were examined, revised and defined to accurately reflect the underlying content.

## 3 Results

A total of 148 participants were included in the study. Of these, 116 were female (78.37%) and 32 were male (21.62%). All participants were undergraduate dental students, with 111 participants from India (75.00%) and 37 (25.00%) participants from Qatar. Twenty‐eight (18.92%) participants were enrolled in Year 2, 16 (10.81%) in Year 3, 45 (30.41%) in Year 4, 9 (6.08%) in Year 5 and 50 (33.78%) were dental interns.

Participants were divided into four age groups, with 3 (2.02%) aged over 30, 8 (5.41%) aged 26–30, 113 (76.35%) aged 21–25, and 24 (16.22%) aged between 18 and 20.

The overall mean score for all items was 1.02 (95% CI 0.82–1.15). Descriptive values for each item are summarised in Table 1. The overall rating was positive, suggesting that, on the whole, CBDE was perceived to be beneficial by the participants.

**TABLE 1:** Descriptive values (all respondents).

Question	Mean	SD (±)	95% CI (lower)	95% CI (upper)
Q1a—I like providing oral health education in the community settings	1.32	0.72	1.20	1.43
Q1b—I enjoy the experience of learning new things in community‐based activities	1.22	0.75	1.10	1.34
Q1c—The course helped me improve my knowledge about the oral health needs of the community	1.20	0.66	1.10	1.31
Q1d—I feel this course prepared me well to provide effective oral health education to the community	1.07	0.75	0.95	1.20
Q1e—This course had a positive impact in my attitude towards clinical preventive oral care	1.18	0.67	1.07	1.29
Q2a—This course helped me improve my communication skills with the community	1.09	0.90	0.95	1.24
Q2b—This course prepared me well to provide oral health education to community members	1.08	0.76	0.96	1.20
Q2c—I was able to establish rapport with members of the community without any difficulty	0.81	0.88	0.67	0.95
Q2d—I feel anxious when engaging with children during my community work	0.30	1.07	0.13	0.48
Q2e—I feel anxious when engaging with adults during my community work	0.36	1.06	0.19	0.54
Q3a—I recognise my professional responsibilities towards the community	1.22	0.59	1.13	1.32
Q3b—I maintain a good professional relationship with all those involved in community activities	1.13	0.66	1.02	1.24
Q3c—The course has motivated me to reflect on my performance	1.11	0.67	1.00	1.22
Q3d—I had good support from my supervisor/teacher	0.95	0.94	0.79	1.10
Q3e—I had good support from my team members	1.17	0.79	1.04	1.30
Q4a—I am able to obtain a comprehensive medical history from patients in primary care settings	0.92	0.71	0.80	1.03
Q4b—I am able to perform a clinical oral examination of patients in primary care settings	1.09	0.63	0.99	1.20
Q4c—I improved my patient management skills through preventive clinical care activities	1.11	0.59	1.02	1.21
Q4d—I am confident of providing clinical preventive care for my patients	1.11	0.68	1.01	1.22
Q4e—Community‐based activities helped me improve my clinical skills in providing chairside preventive care	0.92	0.94	0.77	1.07
Overall	1.02	0.82	0.89	1.15

ANOVA identified significant variation by Age, Country and Stage, with younger students, final‐year students and those studying in Qatar giving more positive responses.

**FIGURE 1:**
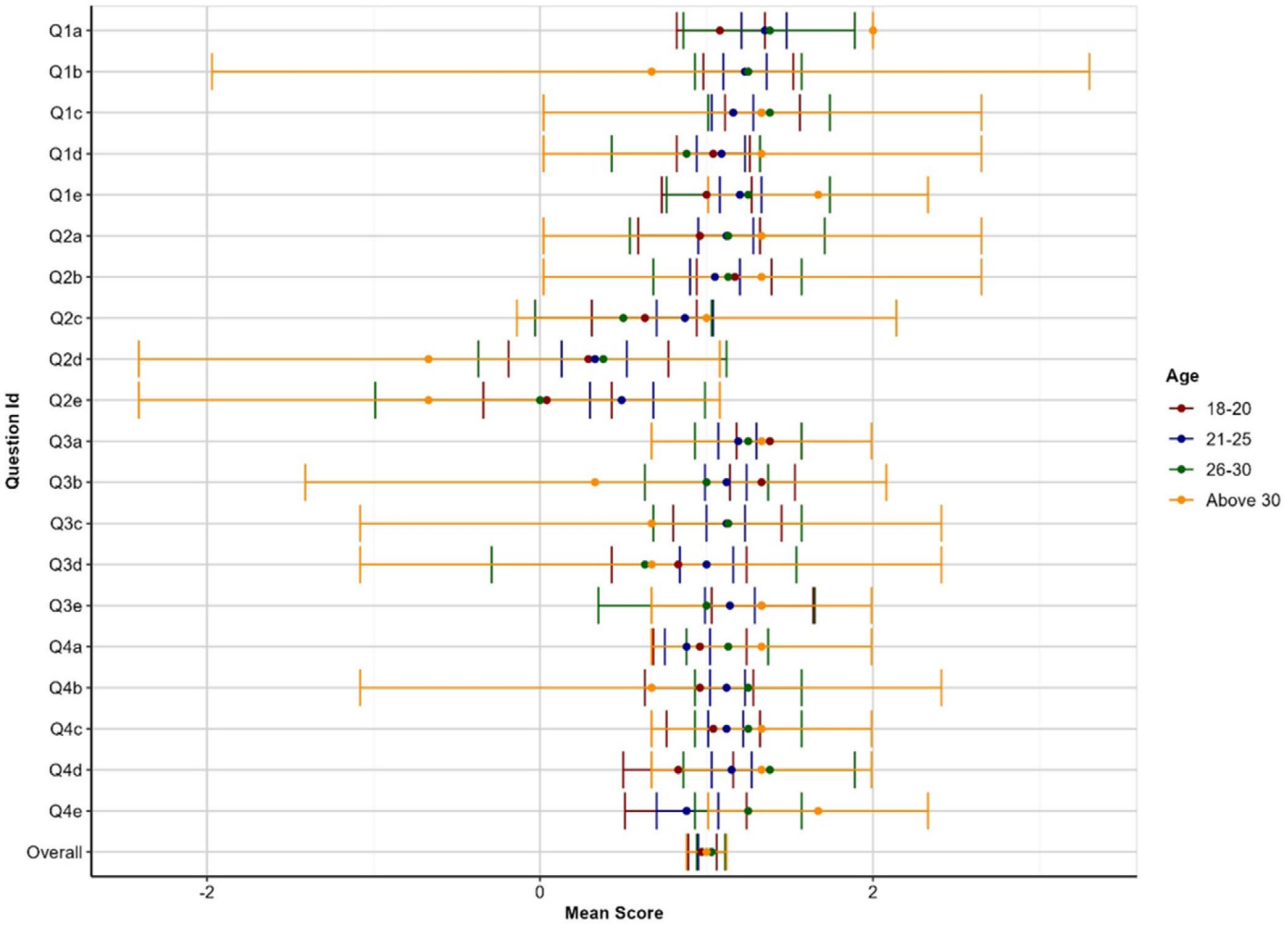
Mean scores with 95% confidence intervals (CI) by age.

**FIGURE 2:**
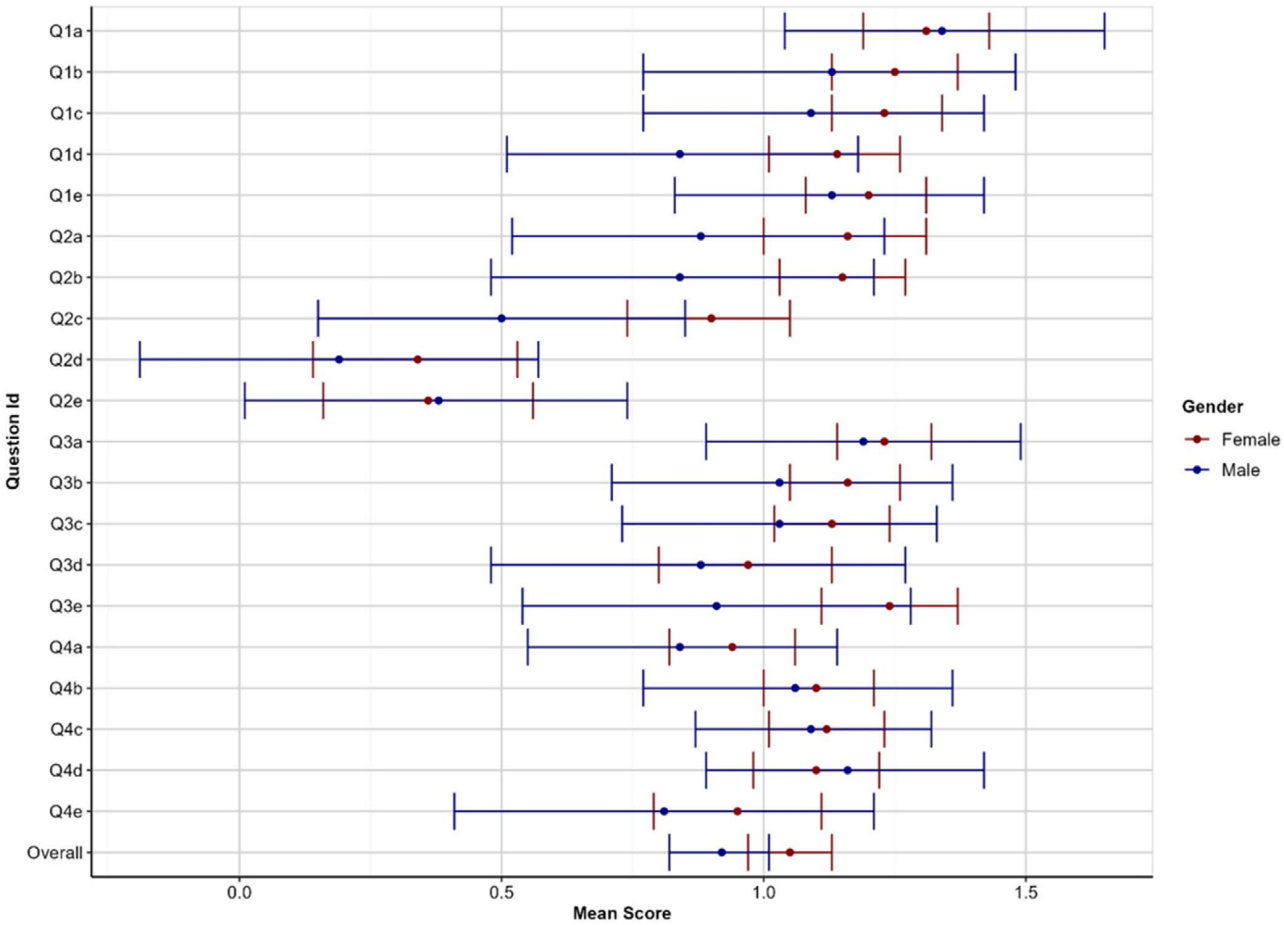
Mean scores with 95% confidence intervals (CI) by gender.

The highest mean score was observed for participants in the 21–25 (1.03 ± 0.81) and 26–30 year (1.03 ± 0.80) age groups. The lowest mean score was recorded for participants in the 18–20 year age group (0.97 ± 0.82). These findings are depicted in Figure 1. The mean score for females was 1.05 ± 0.77 and 0.92 ± 0.98 for males, indicating more positive perceptions and experiences of female participants. However, the differences were not statistically significant (*p* = 0.127). These findings are depicted in Figure 2. The mean scores of participants were compared based on the stage of their education. The results are summarised in Table 2. Dental interns had the highest mean score (1.12 + 0.8), whereas students in Year 2 had the lowest mean score (0.75 + 1.08).

**TABLE 2:** Descriptive values by programme stage.

Question ID	Mean score					Standard deviation (±)					95% CI (lower)					95% CI (upper)				
	Y2	Y3	Y4	Y5	Interns	Y2	Y3	Y4	Y5	Interns	Y2	Y3	Y4	Y5	Interns	Y2	Y3	Y4	Y5	Interns
Q1a	0.68	1.44	1.42	1.44	1.52	1.02	0.51	0.5	0.53	0.58	0.3	1.18	1.28	1.1	1.36	1.06	1.69	1.57	1.79	1.68
Q1b	0.86	1.44	1.36	1.33	1.22	0.93	0.51	0.57	0.71	0.79	0.51	1.18	1.19	0.87	1	1.21	1.69	1.52	1.8	1.44
Q1c	1.07	1.19	1.33	1.33	1.14	0.77	0.66	0.6	0.5	0.67	0.79	0.86	1.16	1	0.95	1.36	1.51	1.51	1.66	1.33
Q1d	0.86	1.06	1.2	1.11	1.08	0.89	0.57	0.69	0.6	0.78	0.52	0.78	1	0.72	0.86	1.19	1.35	1.4	1.51	1.3
Q1e	0.82	1.25	1.36	1.22	1.2	0.82	0.58	0.53	0.67	0.67	0.52	0.96	1.2	0.78	1.01	1.13	1.54	1.51	1.66	1.39
Q2a	0.75	1.06	1.18	1	1.24	1	0.85	0.83	0.71	0.92	0.37	0.64	0.93	0.53	0.98	1.13	1.48	1.42	1.47	1.5
Q2b	0.93	1.25	1.04	1.22	1.12	0.77	0.58	0.82	0.44	0.8	0.64	0.96	0.8	0.93	0.9	1.21	1.54	1.29	1.51	1.34
Q2c	0.25	0.88	0.98	0.67	0.98	1	0.81	0.89	0.87	0.71	−0.13	0.48	0.72	0.1	0.78	0.63	1.27	1.24	1.24	1.18
Q2d	0.21	0.31	0.27	0.33	0.38	1.1	1.2	1.05	1	1.09	−0.2	−0.28	−0.04	−0.33	0.08	0.63	0.9	0.58	0.99	0.68
Q2e	0.07	0.13	0.44	0.33	0.54	0.94	1.09	1.03	1	1.15	−0.28	−0.41	0.14	−0.33	0.22	0.42	0.66	0.75	0.99	0.86
Q3a	1.25	1.19	1.13	1.33	1.28	0.65	0.75	0.66	0.5	0.45	1.01	0.82	0.94	1	1.15	1.49	1.56	1.33	1.66	1.41
Q3b	1.11	1.38	1.07	1.11	1.12	0.74	0.72	0.62	0.33	0.69	0.83	1.02	0.88	0.89	0.93	1.38	1.73	1.25	1.33	1.31
Q3c	1	1.19	1.13	1	1.14	0.94	0.54	0.5	0.5	0.7	0.65	0.92	0.98	0.67	0.94	1.35	1.46	1.28	1.33	1.34
Q3d	0.79	0.69	1.24	1.33	0.78	0.99	1.01	0.77	0.71	1	0.41	0.19	1.02	0.87	0.5	1.16	1.19	1.47	1.8	1.06
Q3e	1.14	1.25	1.29	1.33	1.02	0.93	0.68	0.76	0.71	0.8	0.79	0.91	1.07	0.87	0.8	1.49	1.59	1.51	1.8	1.24
Q4a	0.61	0.94	1.02	1.22	0.94	0.74	1	0.54	0.44	0.74	0.33	0.44	0.86	0.93	0.73	0.88	1.43	1.18	1.51	1.15
Q4b	0.75	1.06	1.22	1.33	1.14	0.8	0.68	0.47	0.5	0.61	0.45	0.73	1.08	1	0.97	1.05	1.4	1.36	1.66	1.31
Q4c	0.79	1.13	1.18	1.22	1.22	0.69	0.81	0.49	0.44	0.51	0.53	0.73	1.03	0.93	1.08	1.04	1.52	1.32	1.51	1.36
Q4d	0.57	1.25	1.18	1.22	1.3	0.84	0.58	0.61	0.67	0.51	0.26	0.96	1	0.78	1.16	0.88	1.54	1.36	1.66	1.44
Q4e	0.46	0.69	0.84	1.33	1.24	1.1	1.3	0.9	0.5	0.62	0.05	0.04	0.58	1	1.07	0.88	1.33	1.11	1.66	1.41
Overall	0.75	1.04	1.09	1.12	1.08	0.93	0.85	0.76	0.68	0.8	0.65	0.95	1.02	1.05	1	0.84	1.12	1.17	1.19	1.16

ANOVA showed a significant impact of age, country and stage of education on the mean scores of the participants (*p* ≤ 0.001) as shown in Table 3. ANOVA identified significant variation by Age, Country and Stage, with younger students, final‐year students and those studying in Qatar giving more positive responses. The estimated marginal means of all questions by demographics are summarised in Table 4.

**TABLE 3:** ANOVA (all questions).

Factor	df	Sum of squares	RSS	AIC	*F*‐statistic	*p*
Age	3	12.96	1931.387	−1249.774	6.642	< 0.001
Gender	1	1.52	1919.946	−1263.362	2.333	0.127
Country	1	11.54	1929.970	−1247.947	17.748	< 0.001
Stage	4	56.88	1975.307	−1185.218	21.87	< 0.001

**TABLE 4:** Estimated marginal means (all questions).

Factor	Level	Responses (*n*)	Adjusted mean
Age	18–20	24	1.27
	21–25	113	1.03
	26–30	8	0.96
	Above 30	3	0.99
Gender	Female	116	1.09
	Male	32	1.03
Country	India	111	0.89
	Qatar	37	1.23
Stage	Year 2	28	0.44
	Year 3	16	1.01
	Year 4	45	1.26
	Year 5	9	1.31
	Interns	50	1.28

### 3.1 Responses to Open‐Ended Questions

*Q.1 Did you experience any problems or barriers (social/cultural) during your community dentistry course? If yes, please explain*.

Responses to this question identified several challenges experienced by the participants during their CBDE learning activities, as summarised in Table 5.

**TABLE 5:** Challenges and barriers in community‐based dental education.

Theme	Sub‐themes	Frequency^a^
**India**		
Inadequate institutional support	Faculty and administration do not provide adequate support to the students to organise their field visits Students do not have access to appropriate resources to deliver oral health education to the communities	++++
Cultural barriers	Some communities do not express interest in oral health education due to conflicts with local customs Language barriers	++++
**Qatar**		
Field visit schedule conflicts with religious obligations	School visits and community activities during the month of Ramadan (fasting) physically draining for the students	+++++
Didactic teaching workload	Didactic teaching content is heavy and time‐consuming Long lectures have a negative impact on student motivation	++++

^a^ Each ‘+’ in the table represents 10% of responses.

*Q.2 Do you have any recommendations to improve the community dentistry courses at your institution?*

The recommendations of participants were focused on addressing the challenges and barriers during CBDE activities unique to their settings. Students in India expressed the need for more field activities and clinical experience in community settings. They also emphasised the need for better logistic support from their institutions to provide adequate resources to the students to enable the organisation and delivery of oral health education in community settings.

Participants from Qatar suggested that the teaching faculty consider their feedback when planning CBDE courses to focus on activities that have a positive impact on student learning, including more field activities and less classroom‐based teaching. They also suggested a mentorship programme to enhance the presentation skills of the students for the delivery of oral health education to the communities.

## 4 Discussion

This study is one of the few to compare CBDE programmes across two countries—India and Qatar—and includes students at different stages of a dental programme. By involving students from both the early and final stages of their studies, the study explores the variations in perceptions and experiences of students with CBDE. The analysis of age‐related differences showed that senior students had the highest satisfaction scores, suggesting that mature students and interns have a better appreciation of the value of CBDE. These findings may be related to the recognition of the importance of interpersonal skills and addressing the expectations of patients from diverse backgrounds in clinical settings. These findings align with the understanding that clinical exposure enhances confidence in and appreciation of CBDE [18, 19, 20]. Studies have also reported that CBDE may provide students with new and formative experiences, allowing them to understand how cultural, educational and economic factors influence the oral health of populations [20, 21]. Gender differences, while not statistically significant, revealed that female participants reported more positive experiences with CBDE, a finding that corroborates previous research on CBDE [11].

This study also uncovered notable geographical and cultural barriers affecting students in India and Qatar. In India, students faced challenges such as inadequate institutional support and a lack of resources, which hindered their ability to deliver effective oral health education. These issues resonate with findings from other studies conducted in resource‐constrained settings, where logistical and financial barriers often limit students' ability to participate in community health activities [22]. Additionally, cultural barriers, including language differences and resistance from communities due to local customs, further complicated students' efforts to implement CBDE effectively.

In Qatar, students encountered different challenges. The physical demands of fasting during Ramadan, coupled with the conflict between community visit schedules and religious obligations, made it difficult for students to participate fully in CBDE activities. This challenge, unique to the cultural and religious context of the region, highlights the importance of adapting CBDE programmes to local circumstances. Additionally, the heavy didactic workload and long lectures were seen as demotivating for students, much like findings in other regions where extensive lecture‐based teaching negatively impacted student motivation [23].

The comparison between India and Qatar underscores the influence of socio‐cultural factors on CBDE implementation. This highlights the need for CBDE programmes to be flexible and adaptable to local socio‐cultural dynamics [23]. Students in India called for more community activities and hands‐on clinical experience, emphasising the importance of practical engagement in community settings [24]. They also stressed the need for better financial and logistical support to facilitate their participation in field activities. In Qatar, students recommended reducing the focus on didactic teaching and increasing fieldwork, along with implementing mentorship programmes to enhance their presentation skills for delivering oral health education.

Based on the findings of this research, a few recommendations can be made to further enhance the learning experiences of students in CBDE. Given the positive impact of learning experiences in community settings, it is important that CBDE is incorporated into undergraduate dental curricula in a spiralling manner. Dental schools may also consider enhancing opportunities for students to gain hands‐on clinical experiences in community settings. Depending on the local circumstances, clinical experience may be provided in dedicated community dental clinics, general dental practices in primary care or the use of mobile dental units in remote locations. Dental institutions should also provide logistic and financial support for CBDE activities to help students in developing educational resources on oral health education. Partnerships with government bodies, non‐governmental organisations (NGOs), charities and local health bodies may enhance the scope and effectiveness of engagement with the communities. One of the key benefits of CBDE is to enhance the cultural competence of dental students through engagement with populations from diverse backgrounds. Dental schools must engage with representatives of local communities to address the socio‐cultural and linguistic barriers for students during their CBDE activities.

There are a few limitations to this study that need to be acknowledged. First, there was asymmetric participation, with three institutions from India and only one from Qatar, potentially introducing selection bias in the comparison between the two countries. Additionally, the response rate was relatively low, limiting the ability to generalise the findings to all dental students in these regions. Future studies should aim for more balanced participation and higher response rates to ensure more reliable and generalisable results. They should focus on multi‐institutional studies comparing CBDE models, especially in different curricula, such as traditional versus problem‐based learning (PBL). Expanding the scope to include multiple institutions and countries would provide a more global understanding of the challenges and opportunities in CBDE. Moreover, a balanced study population across multiple countries would enhance the generalisability of findings and offer more robust insights into how different educational and socio‐cultural environments impact CBDE outcomes.

## 5 Conclusion

The study demonstrates that dental students value CBDE as a crucial component of their education, recognising its benefits in fostering essential skills for effective community engagement. However, it also exposes the challenges faced by students in different socio‐cultural contexts, indicating a need for more localised and supportive frameworks to enhance CBDE experiences. The significant variation in student perceptions based on age, educational stage and country suggests that educational strategies must be adaptable to meet diverse learner needs and contexts.

## Author Contributions

K.A. conceptualised the study, contributed to data collection and drafted the manuscript. S.S., N.G., S.A.A.‐M. and A.A.K. contributed to data collection and drafting of the manuscript. R.G. contributed to data analysis. V.T. contributed to data collection. All authors reviewed the manuscript.

## Ethics Statement

Ethical approval was obtained from the Institutional Review Board of Qatar University (reference number: QU‐IRB 1877‐EA/23, dated 25 May 2023).

## Consent

All participants provided informed consent for publication of this study.

## Conflicts of Interest

The authors declare no conflicts of interest.

## Data Availability

Detailed survey data are available from the corresponding author upon request.
